# Supplementary material for: The causal role of circulating inflammatory markers in osteoporosis: a bidirectional Mendelian randomized study
Source: Front Immunol. 2024 Jul 18;15:1412298. doi: 10.3389/fimmu.2024.1412298 (PMC11291241; doi:10.3389/fimmu.2024.1412298)
Supplement: Supplementary file 1 [file DataSheet_1.zip › supplementary material/CIM-OSTEOPOROSIS /GCST90274836.csv_forest.pdf]

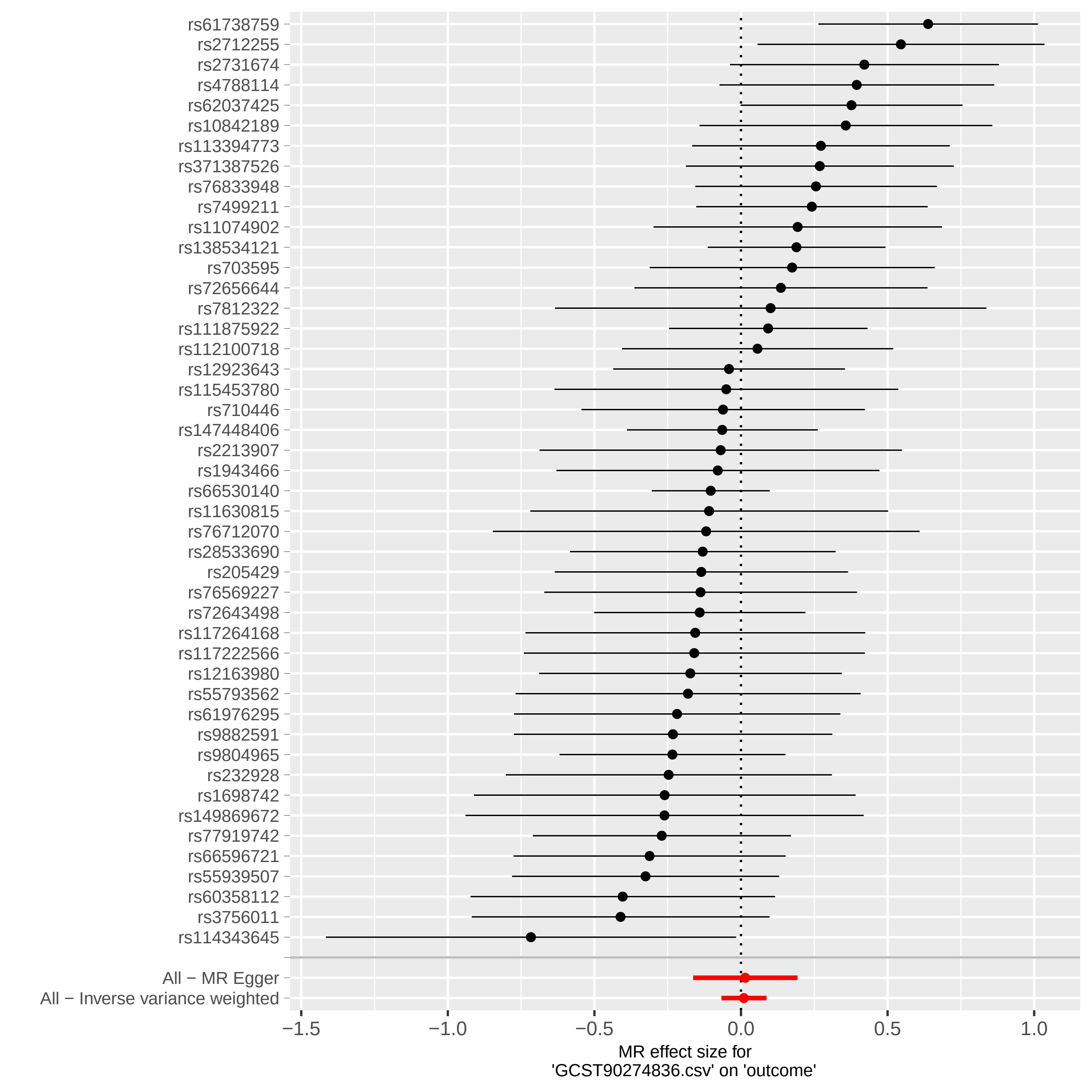
rs61738759
rs2712255
rs2731674
rs4788114
rs62037425
rs10842189
rs113394773
rs371387526
rs76833948
rs7499211
rs11074902
rs138534121
rs703595
rs72656644
rs7812322
rs111875922
rs112100718
rs12923643
rs115453780
rs710446
rs147448406
rs2213907
rs1943466
rs66530140
rs11630815
rs76712070
rs28533690
rs205429
rs76569227
rs72643498
rs117264168
rs117222566
rs12163980
rs55793562
rs61976295
rs9882591
rs9804965
rs232928
rs1698742
rs149869672
rs77919742
rs66596721
rs55939507
rs60358112
rs3756011
rs114343645
All − MR Egger
All − Inverse variance weighted
−1.5
−1.0
−0.5
0.0
0.5
1.0
MR effect size for
'GCST90274836.csv' on 'outcome'
